# Utility of HIV support groups in advancing implementation research in resource-limited settings: experiences from an urban-setting HIV support group in Zimbabwe

**DOI:** 10.1186/s12981-022-00431-w

**Published:** 2022-02-14

**Authors:** Fine Mazambara, Dexter Chagwena, Tinashe Mudzviti, Samantha Sithole, Tsitsi Monera-Penduka, Charles C. Maponga, Gene D. Morse

**Affiliations:** 1 Department of Pharmacy and Pharmaceutical Sciences , International Pharmacotherapy Education and Research Initiative, University of Zimbabwe - Faculty of Medicine and Health Sciences, Avondale, P.O. Box A178, Harare, Zimbabwe; 2 Department of Pharmacy and Pharmaceutical Sciences, University of Zimbabwe, Harare, Zimbabwe; 3 Center for Integrated Global Biomedical Sciences, University at Buffalo, State University of New York, Buffalo, NY USA

**Keywords:** HIV, Nutrition, Retention, Support group, Adherence, Income-generating activities, Psycho-social support

## Abstract

Support groups for people living with the Human Immunodeficiency Virus (HIV) have continued to evolve since their emergence over two decades ago. In addition to providing HIV education and fostering psychosocial support, recent efforts have shifted the focus to socio-economic activities and retention in care. The sense of urgency to adopt new treatment and prevention strategies in sub-Saharan Africa necessitates greater engagement of established HIV care programs, especially among researchers seeking to conduct implementation research, promote prevention strategies and optimize treatment as prevention. To maximize the utility of support groups in doing so, efforts to create an organized, collaborative framework should be considered. This paper aims to describe the process of refocusing an adult HIV peer-support group and illustrate how a structured program was strengthened to sustain implementation research in resource-limited settings, while promoting patient recruitment and retention. A multidisciplinary team of scientists supporting an HIV peer-support group spearheaded the implementation process that authored the successes, challenges and lessons documented over eight years. Psychosocial support, nutrition care and support, adherence education and income generating projects were the main interventions employed. The initiative resulted in seven peer-reviewed publications, submission of 23 scientific abstracts, scientific dissemination at 12 international conferences. Eleven research studies and 16 income generating projects were successfully conducted over eight years. More than 900 patients participated in peer-support group activities every month and 400 were engaged in income generating activities. This multidisciplinary structured program was valuable in the retention and recruitment of patients for implementation research and benefits extended to psychosocial support, microeconomic projects, and improved nutrition. The support group contributed to strengthening implementation research through providing a platform for identification of research priorities, patient recruitment and retention in studies and dissemination of research findings.

## Introduction

An estimated 37.7 million people are living with Human Immunodeficiency Virus (HIV) globally [32]. Sub-Saharan Africa has two thirds of the global population of People Living with HIV (PLHIV) [32]. Zimbabwe has an adult prevalence rate of 12.1% [19]. HIV care and support is needed, combined with HIV treatment, to reduce HIV-related illnesses and deaths and increase the well-being of PLHIV [31]. Support groups are widely accepted as an essential part of HIV care in sub-Saharan Africa and are effective in helping PLHIV manage stress, neutralize stigma and practice new behaviors [9]. Support groups are described as voluntary, small groups, designed for the fulfilment of a common purpose and mutual help and for dealing with a shared problem or condition that alters the normal course of life [7]. Numerous studies have documented the usefulness of support groups in HIV patient care and support [1]. While their role has evolved to provide support in a variety of areas, including life-skills training, performing arts, peer to peer counselling, adherence support and income generating activities [29], there is scarce literature on their usefulness in implementation research. Zimbabwe is among the initial countries to institute formal community-based HIV support systems in sub Saharan Africa during a period when stigma and prejudice were rife [28]. Moreso, nutrition interventions are a pivotal source of support in PLHIV on antiretroviral therapy (ART) in resource limited settings [16].

Support groups provide a base for implementing clinical research through promoting patient recruitment and retention [8]. Despite the long-standing history of support groups in the region, there continues to be lack of standardization across various HIV support groups in terms of structure, activities and the actual definition of support group membership. It is therefore prudent to investigate how support groups can be integrated with academic programs to advance implementation research.

This paper describes how support groups for PLHIV can be useful in supporting implementation research in resource-limited settings, based on a model HIV support group at the country’s largest referral hospital in Harare, Zimbabwe. It outlines a multidisciplinary structured program embedded in a psychosocial HIV peer-support group model to promote patient recruitment and retention, peer-support, facilitate microeconomic initiatives and nutrition education. This paper provides a two-pronged process on how utility of support groups could advance implementation research, based on an observational follow-up of a support group structure. In this context implementation research is defined as the scientific inquiry into questions concerning implementation [26]. It focuses on issues such as identifying implementation problems, understanding factors that hinder or facilitate access to health interventions, developing and testing solutions to tackle implementation barriers, determining the best way to introduce innovations and promoting their large scale use and sustainability [26].

Initially various essential programs to support PLHIV including nutrition assessment and support, psychosocial support, agricultural interventions to address food insecurity, life-skills building initiatives for poverty alleviation and education on medicines and concomitant use with traditional herbs were implemented as part of the PARI Support Group (PSG) program. Implementation factors to document success of these interventions were investigated through a multidisciplinary team of scientists who were part of the PSG overall structure. The second attempt of this paper involves the observational follow-up of the proposed support group model, conducted over a period of eight years. Hence, the purpose of this paper is to demonstrate utility in advancing implementation research in a resource-limited setting.

## Methods

A case study design was employed to document the development and implementation of a PLHIV peer-support group. The observational study was conducted documenting implementation factors and outcomes of a unique support group for PLHIV model for eight years, between 2010 and 2018. The study documented functionality, participation of individual members, activities promoted and conducted in large and small groups. Impact of linking the peer-support group to a multidisciplinary research team were documented. Financial resources supporting the peer-support group, factors influencing sustainability of the group, challenges and successes of the group were also documented. A participatory approach was employed where researchers were part of the multidisciplinary research team that provided clinical and psychosocial support to PLHIV support group members.

### Progress and structure of PARI support group

PARI (Perseverance, Adherence, Responsibility and Integrity) adult HIV support group, was established in 2006. The PARI support group is based at the largest tertiary hospital in Zimbabwe. The HIV outpatient clinic is known as the Parirenyatwa Centre of Excellence and the treatment program is supported by three Ministry of Health partners. The clinic caters for approximately 5000 adults, adolescents and paediatric PLHIV. It provides a comprehensive care model including medical and mental health services, cervical cancer screening, non-communicable diseases screening and treatment, as well as socio-economic support. The support group was established through facilitation by the International Pharmacotherapy Education and Research Initiative (IPERI) as a patient retention and recruitment strategy for a research training program for the International Collaboration on HIV and AIDS Research Training Program. The group continued with funding support from the Fogarty International Center’s AIDS International Training and Research Program (AITRP) through 2016 and subsequently, the HIV Research and Training Program (HRTP). The support group, together with the International Pharmacology Special Laboratory were interlinked, playing a key role in supporting clinical research activities within IPERI. The support group was designed to meet the need for multi-faceted interventions for psychosocial support, patient retention, improved adherence and nutrition outcomes while complementing pharmacology research. PLHIV were recruited into the support group by their peers and volunteer peer counsellors at the Parirenyatwa Opportunistic Infections (OI) clinic. A membership form was completed upon joining the support group.

IPERI assisted PSG in organizing itself and establishing a community advisory committee that monitors clinical research activities at the HIV clinic. A needs assessment was conducted between the support group and the researchers. Identified needs are shown in Table 1.

**Table 1:** Identified needs of PARI Support Group and the IPERI Multi-disciplinary scientist team

PARI Support Group’s needs	IPERI Multidisciplinary Scientists’ Team
Professional psychosocial and peer support	A community advisory committee as a vehicle for community input and monitoring of research studies
Medicines awareness training program	Well informed volunteers for pharmacology studies
Information on drug-herb interactions	Improved access to research and training facilities for investigators
Assured access to ART information	Successful recruitment of patients in HIV pharmacology studies
Nutrition education	Satisfactory patient—retention in HIV pharmacology studies
Kiosk to sell nutritious food Training in self-help projects to improve household income and food security	Well-nourished participants with improved livelihoods

Partnership activities were mutually explored based on these needs. These activities were initiated in November 2006.

### The administrative function

The support group is structured in a way that promotes ownership and maximum participation by PLHIV. The PSG leadership system consists of an executive committee of nine members, elected at the end of each year by the members. The support group is coordinated by a social worker and executive members meet once a month to plan for group activities, share information, resolve conflicts and brainstorm topics for future discussion.

#### Governance system and membership

PSG members’ age ranged from 18 to 76 years. PSG executive provided leadership to the group, coordinating monthly meetings, and identifying members’ needs on a regular basis. The leadership mobilized condolence fees from members in the event of death of a PSG member or close family member. PSG members including peer counselors, trainers, income generating activities project leaders and general facilitators were also part of an organizational structure utilized to discuss research priorities, enroll participants in studies and provide feedback to members. Research priorities identified included the effect of concurrent use of traditional medicine on ART [2, 21, 24], management of HIV comorbidities, HIV and cancer and the long-term effects of treatment. Some of the priorities identified contrasted with researchers’ priorities in that PSG members discussed issues relevant to them.

#### Support group meetings

Support group meetings were held on the first Thursday of each month during lunch hour. The meetings were platforms for group therapy and peer support. During monthly meetings, HIV health information was shared through oral presentations, videos and group discussions led by members of the multi-disciplinary team. Information, education, and communication materials sourced from partner institutions were disseminated during these meetings. Periodically, members completed basic questionnaires to gather information on demography, occupation, nutrition support, ARV regimens and herbal medication use. Refreshments were provided at each meeting when resources were available. In a calendar year, at least 12 formal support group meetings were held. Other meetings included training workshops, support visits, ad hoc meetings to support members in need of assistance, and annual World AIDS Day commemorations. Active support group membership is based on attending at least nine monthly meetings in a calendar year and at least 75% of training workshops and group activities conducted in a year. Support group members met their own travelling costs to meetings and some training workshops unless fundraising had occurred for a specific activity. IPERI researchers continuously engaged with support group members and recruited study participants among PSG and non-PSG members following various protocol designs. Table 2 shows the training workshops that were conducted for PSG members.

**Table 2:** Description of training workshops provided for support group members

Training Activity	Subject Matter	Cumulative Number of Beneficiaries 2011–2018
Life-skills training for income generation	Batik Making, Jewelry, Detergents Liquid Making,	912
Nutrition sensitive agriculture training	Backyard vegetable gardening, Chicken Rearing, Herbal Gardening, Mushroom Production	916
Food Preparation and Preservation	Juice Making, Fruit Jam Making	494
Psychosocial support training to enhance peer support	Ethical use of social media, Psycho-social support training	468
Knowledge on Medicine and Treatment Procedures	Adherence education (Ruzivo Nezvemishonga), ART Treatment, Concomitant Use of Herbal and Traditional Medicines	1080
Nutrition Care and Support Training Sessions	Basic Nutrition Education, Dietary Education	817

^*^Cumulative exceeded active members as non-active members participated. Active membership status was strict as described above

### Financial support

PSG activities were mostly funded from research grants (under IPERI) and further sponsorship from private pharmaceutical companies’ corporate social responsibility. During the first three years (2010–2013), PSG activities were partly sponsored by monthly subscriptions from members, nevertheless payment of subscriptions was not compulsory. Donations started to trickle from the fourth year supporting training workshops, annual World AIDS day commemorations, awareness campaigns, stationery, clothing and food items for vulnerable group members. Table 3 shows external financial support provided to PSG members.

**Table 3:** Financial support provided to PSG activities

Period	Source of funding	Amount (US$)	Number of beneficiaries supported	Purpose of support
2006–2009	PSG Membership Fees	$1200	147	PSG running costs Funds were channeled towards the bereaved, sick, and stranded members
2006–2010	ICHARPT	$3 500	165	Training workshops, annual World AIDS day commemorations, stationery
2006–2018	Private Companies; HETERO, PCD, New Avakash	$12 000	227	Training workshops, annual World AIDS day commemorations, awareness campaigns, stationery, clothing and food items for vulnerable group members
2011–2016	Aids International Training and Research Program (AITRP)	$12 500	167	Training workshops, annual World AIDS day commemorations, Conference Attendance and awareness campaigns
2017–2021	HIV Research and Training Program (HRTP)	$20 596	227	Training workshops, refreshments and bus fare reimbursements for meetings

### A multidisciplinary scientific team providing support to PSG

**Fig. 1:**
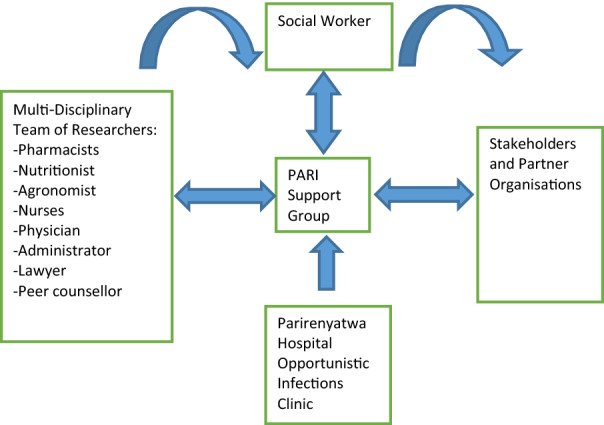
Organizational structure of the PARI Support Group

The support group was in close collaboration with a multi-disciplinary team which includes: social workers, physicians, pharmacists, nurses, nutritionists, legal practitioners and agricultural advisors to meet the needs of the group (Fig. 1). The PSG was also affiliated with two academic institutions: University of Zimbabwe, Department of Pharmacy and Pharmaceutical Sciences and the University at Buffalo School of Pharmacy and Pharmaceutical Sciences whose graduate students, fellows and resident scientists were engaged in implementation research.

### Social worker role

The social worker served as a liaison between patients, IPERI research work and the inter-disciplinary scientists to ensure patient wellness. Retention of participants to studies was facilitated for by the social worker. The social work role extended to supporting PLHIV with psychosocial support, capacities for problem solving, life-skills to cope and providing links to the national social welfare program and community resources. Such social work intervention was essential in providing a holistic approach to long-term patient retention and participation in clinical research.

### Key activities engaging PSG members between 2011 and 2018

Key activities engaging PSG members between 2011 and 2018 include members’ training in income generation, psychosocial peer-support, nutrition support and improved health education. More than 400 members benefitted from various training programs provided annually over the eight-year period (Table 2).

### Nutrition assessment, care and support for PSG members

Routine nutrition care and support was provided by nutrition scientists. Support group members received group nutrition education and individual nutrition counseling for members with deteriorating nutrition status. Nutritionists provided education and linkages to clinic and community-based nutrition programs to avoid introducing a parallel system to the government nutrition program at the Parirenyatwa OI clinic. Routine nutritional assessment was conducted by the clinic. A onetime assessment was conducted in 2014 to provide an overall picture of nutritional status of PSG members.

As nutrition plays a crucial component for PLHIV, routine nutrition education, care and support was a core component for PSG. Members were educated on basic nutrition requirements, healthy foods and received nutrition counseling. Since PSG was linked to the Parirenyatwa OI Clinic routine nutrition assessment and support was not conducted as part of PSG activities but provided as part of the OI clinic’s routine services to encourage clinic attendance. Weight was measured at each clinic visit and height was measured once a year based on the national guidelines for treatment and management of PLHIV. Malnourished patients were referred to a United Nations World Food Program Nutrition Support for Antiretroviral Therapy (NSART) program implemented at Parirenyatwa OI clinic during this period of the study. The NSART program was implemented in HIV clinics through local partners around the country. This specific program at Parirenyatwa OI clinic was implemented by Adventist Development and Relief Agency (ADRA) Zimbabwe.

The NSART program was an approach to deliver nutrition services (assessment, education, counseling and food provisions) for pre-ART and ART patients who were malnourished. Patients in the NSART program received corn-soya blend and were given a food voucher which they could redeem at specific retail supermarkets in their residential communities. Additionally, households with malnourished patients received maize-meal, beans and vegetable oil for the family through this program. Therefore, the NSART program provided comprehensive nutrition support for the PARI support group as malnourished members would receive assistance from this organization while developing sustainable life skills to help their households become food-secure in the long-term. Information on the use of traditional medicines and presence of comorbid conditions was also retrieved from everyone’s health record and during nutrition support individual counseling sessions (ADRA NSART Report; 2016).

An evaluation of nutritional status was conducted among adult PLHIV participating in the PSG and attending Parirenyatwa OI clinic between April and September 2014. This was a one-time assessment to describe the nutritional status of PSG members. Any member attending a support group monthly meeting during these six months had their nutritional status evaluated and recorded. Nutritional assessments were conducted by a nutritionist or nutrition intern during each meeting and the individuals notified of their Body Mass Index (BMI).

## Results

**Fig. 2:**
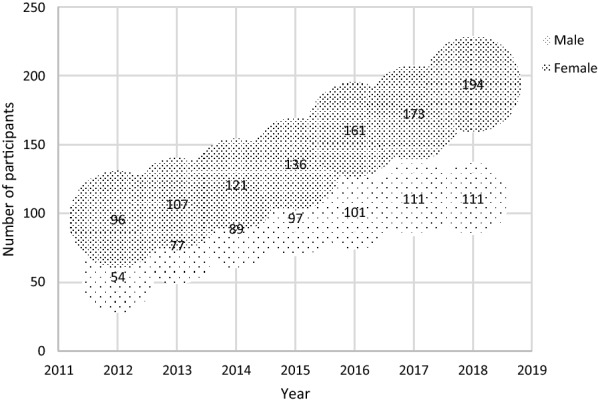
Support group membership from 2011 to 2018

A disproportionate increase in PSG membership was observed during the eight-year period under review (Fig. 2). Members doubled between 2011 and 2018.

### PARI support group participation in research

Approximately 60% of PARI support group members were actively involved and attended each monthly meeting, with an average of twelve new members joining each month. Researchers recruited support group members with ease. Retention of study participants was necessitated by routine support group follow up processes. The IPERI researchers’ projects were mainstreamed into the support group’s activities and patients were interested in research dissemination meetings conducted by researchers. As of December 2018, 23 international abstracts were produced and 12 international conferences were attended. PSG members supported research that produced seven peer reviewed publications and abstracts in areas of nutrition [3] and pharmacology [2, 20–25]. Participants in the first regulatory approved clinical study of herbal medicine included PSG members [20]. The clinical study followed surveys which had been also done among support members which highlighted the extent and patterns of herbal medicine use that was not disclosed to clinicians and the need for clinical safety data [3, 22]. The protocols for these studies included a patient recruitment plan that would be implemented through PSG. Potential participants were sensitized to study protocols at PSG meetings and recruitment was initiated at the same.

In addition to this, PSG has been involved in eight annual World AIDS Day commemorations. Each year an average of 120 (s.d = 20.7) members attended the annual World AIDS day commemorations between 2011 and 2018. PSG was represented at two international AIDS conferences in 2012 and 2014, showcasing the impact of microeconomic activities for PLHIV as a patient retention strategy in resource limited settings. Other achievements are shown in Table 4.

**Table 4:** Achievements of PSG members and Multi-disciplinary team members

	Achievements	Description		Notes/ Comments
		Patients	Researchers	Details
1	Publications	0	7	PSG members participated in 7 studies that were published in peer reviewed journals
2	Abstracts Submissions	3	20	23 Abstracts were submitted to International conferences
3	Dissemination at an International Conference	3	9	-IAS 2011, Rome, Italy -AIDS2012, Washington DC -IAS 2013, Cape town, South Africa -ICASA2013, Kuala Lumpur, Malaysia - ZINC 2013, Harare, Zimbabwe -AIDS 2014, Melbourne, Australia -ICASA2015, Zimbabwe -ETA 2015, Harare, Zimbabwe -WHI, 2016
4	Grant Renewal	0	1	2016 HRTP grant renewal
5	Undergraduate Honors projects	n/a	2	-Traditional medicine -Nutrition and HIV
6	Doctoral projects conducted	n/a	4	-PSG members participated in 4 doctoral projects
7	Post-doctoral projects conducted	n/a	2	PSG members participated in 2 post-doctoral researches as subjects/participants
8	Training workshops conducted	8	n/a	Batik making, Marmalade Jam making, juice making, backyard gardening, herbal gardening, chicken rearing, Psycho-social Support, ethical use of social media trainings was conducted for the patients
9	Income generating projects initiated	16	n/a	16 projects were initiated following training workshops
10	MOUs signed	1	0	Office of the President and Cabinet department for psychomotor activities in education
11	International Commemorations held	8	8	World AIDS Day commemorations were done annually for both researchers and patients

IAS-International AIDS Society

ICASA-International Conference on AIDS and STIs in Africa

ETA – Evidence to Action

### Key activities that supported implementation research

The support group facilitated activities that supported implementation research. Key activities include identification of research priorities, recruitment of research participants, retention of participants in studies and dissemination of research findings to community groups.

#### Nutrition assessment, care and support

A rapid nutrition assessment was conducted in 2014 among PARI support group members coinciding with a cross-sectional national survey focused on nutrition assessment and vulnerability profiling of PLHIV enrolled at OI/ART clinics in Zimbabwe [17]. Nutritional status of PSG members from the 2014 rapid assessment were compared to nutritional status of PLHIV attending OI clinics based on a nationally representative survey conducted during the same period, between April and September 2014 [17]. In this paper data from the 2014 national survey on nutrition assessment and livelihood profiling of adult HIV study was utilized and compared. This cross-sectional national survey included participants older than 15 years. Similar weight and height anthropometric measurements were used to classify BMI. The sample size for the national assessment survey was 1, 420 patients using a design effect of two, a 95% confidence interval, a 2.5% margin of error and an 80% response rate. The estimate point was prevalence of malnutrition among HIV-infected adults estimated at 10.3% [17].

The 2014 PSG nutritional evaluation was a cross-sectional rapid assessment conducted between June and September 2014. Members had their nutritional status assessed once during the four months and recorded. An interviewer administered questionnaire was used to ask questions on factors influencing their nutritional status such as household income, chronic illness, medication and traditional medicine use, food security including other measures. Findings on the nutritional status have not been reported elsewhere. Height, weight, waist and hip circumference were measured using SECA adult scale, height board and a tape measure respectively. To estimate the nutritional status of PSG members a minimum sample size of 240 participants were required using a design effect of 2, a 95% confidence interval, a 2.5% margin of error and estimated prevalence of wasting of 10% among PLHIV based on previous sub-Saharan studies. Body mass index (BMI) was calculated from height and weight. Waist and hip circumferences were measured to classify malnutrition according to WHO standards. Data was analyzed using STATA to estimate prevalence, and chi-square test employed to test for association. A p-value < 0.05 indicating a statical significant difference.

A total of 244 PSG members with a median age of 38 (IQR; 31–44) years had their nutritional status evaluated. Eighty-three percent earned less than USD $200 as monthly income. Of those who were on ART, 76% of participants were taking a non-nucleoside reverse transcriptase inhibitor-based regimen while 15% were on a protease inhibitor based regimen. Use of cotrimoxazole (an antibiotic used for prophylaxis against Pneumocystis pneumonia (PCP) and Toxoplasmosis gondii in HIV-infected individuals) was reported by almost two-thirds of participants. Only twelve participants reported use of traditional medicines.

**Fig. 3:**
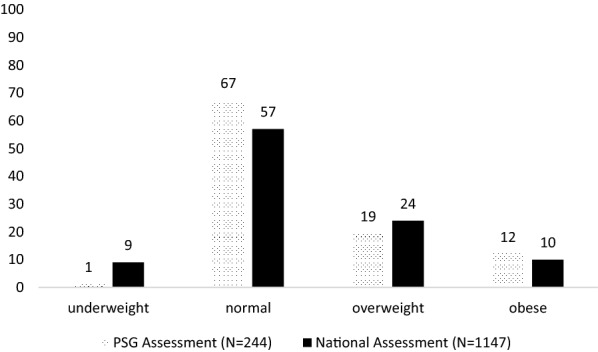
Nutritional status of PSG adult members attending PARI Support Group in 2014

Prevalence of undernutrition (BMI < 18.5) was low among PSG members (1%) compared to a national HIV adult population of 9%. Overweight (BMI > 24.9) was also low among PSG participants (19.1%) compared to the national HIV adult population (24%) while obesity (BMI > 29.9) was high among PSG members (12%) (Fig. 3) [17]. Waist-hip ratios indicated that more men (65%) compared to women (29%,p < 0.05) were at substantially increased risk for obesity- related non-communicable diseases (NCDs) that include type 2 diabetes mellitus, hypertension and cardiovascular disease. Twenty-two percent of the participants suffered from at least one NCD and this was associated with their nutritional status (p < 0.05). The median duration on ART was four (IQR; 2–6) years and this was not significantly associated with members’ nutritional status.

#### Innovations to support implementation

A virtual support group was established on the WhatsApp application and Short Message Service (SMS) platforms. This initiative helped to reach patients who had busy schedules, the sick, and those who could not attend the face to face support group meetings due to other reasons. The virtual support group was established in 2017 in response to the decentralization of ART and “treat all recommendations” that had been rolled out by the Ministry of Health and Child Care [18]. Due to decentralization some members could no longer travel to Parirenyatwa Central Hospital to attend support group meetings as they had been decentralized to their local clinics and groups. The virtual support group also reached PSG members who had busy schedules due to work or family commitments including those who moved to other areas outside of Harare. As of December 2020, the WhatsApp platform had 121 members including one social worker and eight researchers. The virtual support group offered psychosocial support for PLHIV. It was also instrumental in the recruitment and retention of study participants. Researchers used the platform to inform PLHIV about the study and ask any questions pertaining to their studies. The virtual support group was also used as a platform for dissemination where researchers presented results to patients.

## Discussion

This case study is an exemplary model which incorporates elements necessary for advancing implementation research and HIV patient care and support concurrently. Collaboration between the multi-disciplinary team of scientists and patients proved to be valuable in meeting the needs of patients and researchers alike. Reaching planned targets for recruitment in a clinical study is crucial, so as retention of participants in the study [10]. Researchers who recruited participants from PSG reported easy implementation of study protocols and cooperation of study participants. This is in line with existing literature suggesting that face to face recruitment of study participants leads to highest yield in study participants [4]. Also, regular feedback from researchers is known to improve retention of patients in research studies [30].

Attaining a high level of participation in such activities within the current structure ideally provides a base from which to conduct and monitor clinical outcomes. The assumption is that the more engaged and financially independent PLHIV are, increased knowledge and support is shared among them, which ultimately results in good clinical outcomes and improved adherence to ART. However, this cannot be determined without incorporating objective methods to assess adherence [23].

Of concern, was the disproportionate increase in membership of males in comparison to female counterparts. This is consistent with findings from other studies [11, 15, 33]. Increased participation of men in HIV support groups plays an important role as they are safe spaces in which men share experiences with their peers, receive peer support, work on their emotions and access peer counseling for grief and anger (CRS, 2012). More so, there has been an increased need to form gender-specific support groups, different designated meeting days and more flexible designs to improve male participation [12, 13, 15].

Previous studies on peer support have not consistently examined nutrition aspects in support groups [1]. In this case study we noted that, along with other benefits to PLHIV, the role of good nutrition in HIV care has been well demonstrated, hence the importance of ensuring that PLHIV receive adequate nutrition care and support [34]. In this paper PSG members had nutritional status comparable to the nutritional status observed among the PLHIV in Zimbabwe. Proportion of members with malnutrition such as underweight and overweight was very low compared to the national adult HIV population, although this study could not determine if this difference was significant. Routine nutrition education and counseling was provided as part of the PSG activities and could have contributed to improved nutritional status among PSG members, although this could not be confirmed due to the design of our study that was a cross-sectional assessment. Food security and nutrition have been documented as major factors affecting adherence to ART, as patients without access to proper nutrition tend to skip medication [6]. Through life-skills training, nutrition education and counseling, PSG members received support to adopt improved dietary patterns and access to diverse foods. Improved nutritional status of PSG members could have been a result of improved household food security, increased access to nutritious foods through backyard gardening and buying power realized from income-generating activities.

However, levels of obesity among HIV-patients were of concern and PSG members were not spared from this public health threat. Increased levels of overnutrition has also been reported in other national studies among the Zimbabwean population [17, 37]. Overnutrition among PLHIV has become more significant and nutrition interventions need to shift from an emphasis on undernutrition, to addressing both forms of malnutrition [14, 35]. Therefore, there is a need for more rigorous studies to evaluate impact of support groups on both forms of malnutrition among PLHIV on ART in resource-limited countries. Nutrition education can assist PLHIV in various settings to access and make nutritious, affordable, and culturally appropriate food choices. Nutrition counselling has been upheld to increased nutrition knowledge of PLHIV [36].

Use of technology in research enables practitioners to reach more clients in diverse communities [5, 27]. The PARI virtual support group was well suited to the technological advances and ART decentralization experienced in the country during this period and proved valuable in achieving some of the desired goals.

## Future considerations

Following the successful implementation of a multi-disciplinary approach, prospects of PSG include re-inventing the support group in line with recent advances in HIV care and treatment. Regarding technology, exploring other social networks and new research opportunities that can be generated from PSG is warranted. Integrating the virtual support group to existing web-based databases for routine HIV patient data is also crucial. As the support group is growing, there is increased need to secure funding and generate independent source of income for continuity and further growth of the group.

## Limitations

This study examined only one peer support group from an urban setting hence findings may not be generalizable to other settings. As well, the proposed model does not fit in low-income rural and peri urban areas where patients cannot afford their own travel costs to attend some of the support group meetings. Funding should be sought to cover transport costs for attending all support group meetings and trainings for the mutual benefit of both researchers and PLHIV. This study did not look at the patients who were receiving HIV treatment at Parirenyatwa hospital, but not part of the PARI group, therefore the study lacked comparison of the treatment failure and adherence in patients in the support group with those who were not in the support group. The study also lacked information about the longitudinal analysis of retention or drop-out in association with support group interventions. Also, this study was carried out by the organization facilitating the service.

## Key Highlights resulting from this experience

Key highlights resulting from this experience is the usefulness of adult support groups in advancing implementation research mainly through patient recruitment and retention in studies, identification of research priorities and facilitating for dissemination of research findings. Another highlight from this experience is the integration of support groups and academic programs to advance implementation research.

## Conclusions and recommendations

Adult HIV support groups can potentially advance implementation research while improving nutrition care and support, psychosocial wellbeing, and adherence to ART. Such a comprehensive approach does provide the much-needed support to implementation research. This mutual partnership can be replicated in similar contexts in furthering implementation research.

Further research is warranted to determine preferred format and timing of peer support groups, optimum number of support group members and other allied health care practitioners that can be included in the multi-disciplinary model.

## Data Availability

The datasets used and analyzed during the current study are available from the corresponding author on reasonable request.
